# Distinct Defects in Marginal Zone B Cells and Filtration Function Characterize Hyposplenism in Persons With HIV-1 on Prolonged ART

**DOI:** 10.1093/ofid/ofaf644

**Published:** 2025-10-14

**Authors:** Ségolène Debiesse, Anne-Sophie Bedin, Amandine Pisoni, Matthéo Alcaraz, Joseph Fouchet, Nathalie Pansu, Corine Merle de Boever, Alain Makinson, Edouard Tuaillon

**Affiliations:** Pathogenesis and Control of Chronic Infections, University of Montpellier, INSERM, Etablissement Français du Sang, Montpellier, France; Pathogenesis and Control of Chronic Infections, University of Montpellier, INSERM, Etablissement Français du Sang, Montpellier, France; Department of Virology, Montpellier University Hospital, Montpellier, France; Pathogenesis and Control of Chronic Infections, University of Montpellier, INSERM, Etablissement Français du Sang, Montpellier, France; Department of Virology, Montpellier University Hospital, Montpellier, France; Department of Infectious and Tropical Diseases, Montpellier University Hospital, Montpellier, France; Department of Infectious and Tropical Diseases, Montpellier University Hospital, Montpellier, France; Department of Infectious and Tropical Diseases, Montpellier University Hospital, Montpellier, France; Pathogenesis and Control of Chronic Infections, University of Montpellier, INSERM, Etablissement Français du Sang, Montpellier, France; Department of Virology, Montpellier University Hospital, Montpellier, France

**Keywords:** HIV infection, hyposplenism, pneumococcal disease, splenic function

## Abstract

**Background:**

Persons with HIV (PWH) are at increased risk of pneumococcal disease. This study explored filtration and immune function of the spleen, investigating predictors of hyposplenism in PWH on prolonged antiretroviral therapy (ART).

**Method:**

We developed a flow cytometry protocol to enumerate Howell-Jolly bodies (HJBs) and marginal zone B cells (MZBs), comparing results from splenectomized patients and healthy donors. We then analyzed HJBs and MZBs in a cross-sectional study to investigate spleen impairment in 96 PWH on prolonged ART.

**Results:**

Based on results obtained from asplenic patients and healthy controls, we determined optimal cutoff values of 0.05% for HJBs and 10% for MZBs; these allow accurate differentiation between the 2 groups. HJB results were well correlated with microscopic enumeration of HJBs and pocked red blood cells, considered reference methods for spleen filtration. Among 96 HIV patients on prolonged ART, 9.4% exhibited impaired splenic filtration (HJB >0.05%), associated with a history of AIDS. Older HIV patients showed a higher frequency of HJBs, though only 1 exceeded the pathological threshold. MZBs were significantly decreased in 59.4% of HIV participants, with no correlation between MZB depletion and presence of HJBs.

**Conclusions:**

These findings highlight that PWH exhibit depletion of splenic marginal zone B cells, and some demonstrate splenic filtration dysfunction, reflecting persistent splenic impairment despite extended viral suppression. Further research is needed to understand the long-term impact of HIV on splenic function and to develop strategies to improve clinical outcomes in this aging population.

The spleen plays a pivotal role in immunity, with 2 primary functions localized in distinct anatomical compartments: blood filtration by the reticuloendothelial system in the red pulp and antibody production by follicles in the white pulp. Filtration function involves the clearance of opsonized bacteria and parasitized red blood cells by macrophages, while immunoglobulin (Ig) M+/IgD+ memory B lymphocytes, known as circulating marginal zone B cells (MZBs), are crucial for producing antibody against encapsulated bacteria [1].

Functional hyposplenism is common during HIV-1 (HIV) infection. Impaired splenic filtration was reported in AIDS patients as early as 1985 [2]. Before the onset of combined antiretroviral therapy (ART), hyposplenism was observed in approximately one-third of persons with HIV (PWH) [3]. Additionally, depletion of IgM memory B cells has been documented in untreated HIV patients [3]. HIV-associated hyposplenism correlates with the clinical stage of the disease [4]. Antiretroviral therapy appears to only partially restore the MZB (IgM/IgD memory B cells), with some HIV patients keeping cell counts as low as those in splenectomized individuals [5–8].

The clinical implications of hyposplenism in HIV-infected individuals are significant. Reduced splenic function and diminished IgM response to polysaccharide antigens contribute to the vulnerability to infections caused by encapsulated bacteria such as *Streptococcus pneumonia* and *Haemophilus influenzae*. Direct epidemiological evidence supports the association between hyposplenism and an increased risk of invasive pneumococcal disease. In the study by Chong et al. [9], the incidence rate of overwhelming infection in hyposplenic patients was 9.86 per 1000 patient-years, with *Streptococcus pneumoniae* responsible for most documented cases.

Moreover, a study has reported an association between increased mortality and impaired spleen function in HIV-infected children with malaria [10]. The risk of invasive pneumococcal disease was estimated to be 46 to 100 times higher than in the general population during the early 2000s [11–13]. The incidence of invasive pneumococcal disease is particularly high when the CD4 count falls below 200/mm^3^. However, in a Dutch national study, incidence rates of invasive pneumococcal disease and community-acquired pneumonia remained higher than in the general population in PWH with CD4 levels >500/mm^3^ (946 vs 188 per 100 000 person-years of follow-up) [13]. This study identified older age, CD4 count <500/mm^3^, smoking status, and chronic obstructive pulmonary disease as risk factors for community-acquired pneumonia in PWH. Despite effective ART, recent studies have confirmed that the incidence of invasive pneumococcal disease remains substantially elevated in PWH compared with the general population, with adjusted risks reported to be >7-fold higher [13, 14].

Although ART and vaccination have reduced the incidence of invasive pneumococcal disease in PWH, it remains substantially higher than in the general population [12, 15–17]. Some immune dysfunctions persist even after long-term ART-mediated viral suppression and CD4 recovery [15], and it remains uncertain whether hyposplenism can be fully reversed with prolonged ART. Moreover, structural changes in the spleen occur with aging, leading to diminished functions of this organ [16]. As the median age of PWH regularly increases in high-income countries [17], the frequency of hyposplenism could rise in this population.

Diagnosing hyposplenism is challenging due to the lack of straightforward, noninvasive, and sensitive tests. Traditionally, the detection of Howell-Jolly bodies (HJBs), which are erythrocytes containing nuclear remnants, has been used to assess splenic filtration function. The enumeration of pocked red blood cells (pocked RBCs)—erythrocytes containing vacuoles [18]—offers better sensitivity and can be considered the gold standard for detecting impaired splenic filtration capacity [19]. Both HJBs and pocked RBCs reflect the spleen's ability to filter out defective erythrocytes and have been shown to correlate with the degree of splenic dysfunction [20]. However, the enumeration of HJBs and pocked RBCs relies on labor-intensive techniques that limit their use in routine diagnostics, and these markers assess only the red pulp's filtration function. They do not reflect immune protection against encapsulated bacteria mediated by the white pulp, in particular through IgM+/IgD+ memory B lymphocytes of the marginal zone.

Analysis of spleen function in PWH is crucial to better our understanding of the evolution of splenic function in PWH with controlled disease with prolonged ART and to guide prophylactic measures to mitigate infection risks in this population. In this study, we developed and validated a flow cytometry–based assay to assess both splenic filtration (via HJBs) and immune function (via MZBs). We hypothesized that functional hyposplenism persists in a subset of PWH despite long-term viral suppression and immune recovery on ART and that distinct mechanisms may differentially affect the red and white pulp functions of the spleen. This study aimed to characterize the prevalence and nature of splenic dysfunction in virally suppressed PWH and to identify clinical predictors of persistent hyposplenism in this population.

## METHODS

### Design of the Study

We developed a cytometry protocol to enumerate HJBs and validated the results by comparing them with reference methods: HJB and pocked RBC enumeration by microscopy. Then, we analyzed HJBs and MZB cells in a cross-sectional study to investigate spleen impairment in PWH on prolonged ART. Patients were stratified into 2 groups based on their age, below or over 70. The age of 70 was chosen as a clinically relevant threshold to define advanced age, in line with a recent longitudinal study analyzing frailty dynamics in PWH, including patients from our hospital [21]. Healthy controls and splenectomized patients were used for comparison.

### Patients and Healthy Donors

This study was conducted at the University Hospital of Montpellier. Blood samples were collected using EDTA tubes. PWH followed as outpatients were selected randomly or based on history of invasive pneumococcal disease. Patients with detectable HIV viremia in the last 12 months were excluded. The study was approved by our institutional review board (DRI-2022-70). The control groups consisted of 20 asplenic patients from the French national cohort SPLEEN (NCT04199403 SPLEEN), followed in the same center, and 13 healthy volunteers (blood donors). Characteristics of healthy controls, asplenic participants, and HIV patients are summarized in Table 1.

**Table 1.** Patient Characteristics

	Healthy Controls	Asplenic Patients	HIV Patients
No. of patients	13	20	96
Age, median (IQR), y	40 (26–46)	61 (50–97)	55 (47–64)
Sex ratio M/F	6/7	11/6	72/24
Duration of HIV viral suppression, mean (95% CI), y	…	…	11.5 (10.2–12.8)
Invasive pneumococcal disease	…	…	4

Abbreviation: IQR, interquartile range.

### HJB Flow Cytometry Quantification

Blood (50 µL, diluted 1:100 in phosphate-buffered saline [PBS]) was stained for 15 minutes at room temperature with a cocktail consisting of 20 µL of anti-CD235a (GpA) FITC (clone HIR2, Biolegend) diluted 1:500 in PBS, 5 µL of anti-CD71 PE (clone M-A712, Becton Dickinson), 5 µL of anti-CD45 APCH7 (clone 2D1, Becton Dickinson), and 22 µL of Hoechst 33342 (H3570, Invitrogen) diluted 1:1000 in PBS. The mixture was gently pipetted to mix. Cells were washed with 2 mL of PBS and then resuspended in 550 µL of PBS. A minimum of 300 000 erythrocytes (CD235a+, CD45–) were acquired for each sample. Negative tube and Fluorescence Minus One (FMO)–negative controls for CD71 and Hoechst expression were included in each experiment. Acquisition was performed on a 3-laser, 10-color Navios flow cytometer, and analyses were conducted using Kaluza Software, version 2.1 (both from Beckman Coulter, Brea, CA, USA).

### Marginal Zone Memory B Lymphocytes Flow Cytometry Quantification

Blood (100 µL) was stained for 15 minutes at room temperature with a cocktail consisting of 5 µL of anti-CD19 BV421 (clone HIB19, BioLegend), 3 µL of anti-CD27 PC5 (clone 1A4LDG5, Beckman Coulter), and 5 µL of anti-IgD FITC (clone IA6-2, Beckman Coulter). Red blood cells were lysed using FACS Lysing Solution 1× (BD Biosciences). Cells were washed 3 times with PBS and then resuspended in 200 µL of PBS. A minimum of 2000 B cells (CD19+) were acquired for each sample. Negative tube and FMO-negative controls for CD27 and IgD expression were included in each experiment. Acquisition was performed on a 3-laser, 10-color Navios flow cytometer, and analyses were conducted using Kaluza Software, version 2.1 (both from Beckman Coulter, Brea, CA, USA).

### Howell-Jolly Bodies Visualization and Quantification

For each healthy donor and asplenic patient, HJBs were visualized using microscopy on peripheral blood smears stained with May-Grünwald Giemsa. HJBs were manually counted within a total of 5000 erythrocytes by 2 examiners, and percentage of HJBs was calculated.

### Pocked RBC Quantification

Pocked RBCs (intraerythrocytic vacuoles) were visualized in whole blood diluted 1:50 in AIM-V buffer using Differential Interference Contrast (DIC) microscopy. Images were captured with a Zeiss AxioImager Z2 microscope equipped with a 100× oil immersion objective. The percentage of pocked cells was calculated per 500 erythrocytes.

### Statistical Analysis

Data were analyzed using GraphPad Prism software (version 7.00) and compared using the Mann-Whitney *U* test (2-tailed) or Kruskal-Wallis test. Areas under the curve (AUCs) were calculated along with their 95% CIs. Cutoffs for HJBs and MZBs were determined using receiver operating characteristics (ROC) curves. Assay agreement between HJB enumeration methods and HJBs and pocked cells was also assessed with Cohen's kappa (<0.21, poor; 0.41–0.60, slight; 0.61–0.8, substantial; >0.81 almost perfect).

Correlations were assessed with Spearman's correlation test. A *P* value of <.05 was considered significant. Only 1 variable was normally distributed, as tested by the Kolmogorov-Smirnov test. Mean ages in each group were compared using a *t* test, and other continuous variables were compared using the Mann-Whitney *U* test (GraphPad Prism software, version 7.00).

## RESULTS

### Validation of a Flow Cytometry Method for Quantification of HJBs

**Figure 1.**
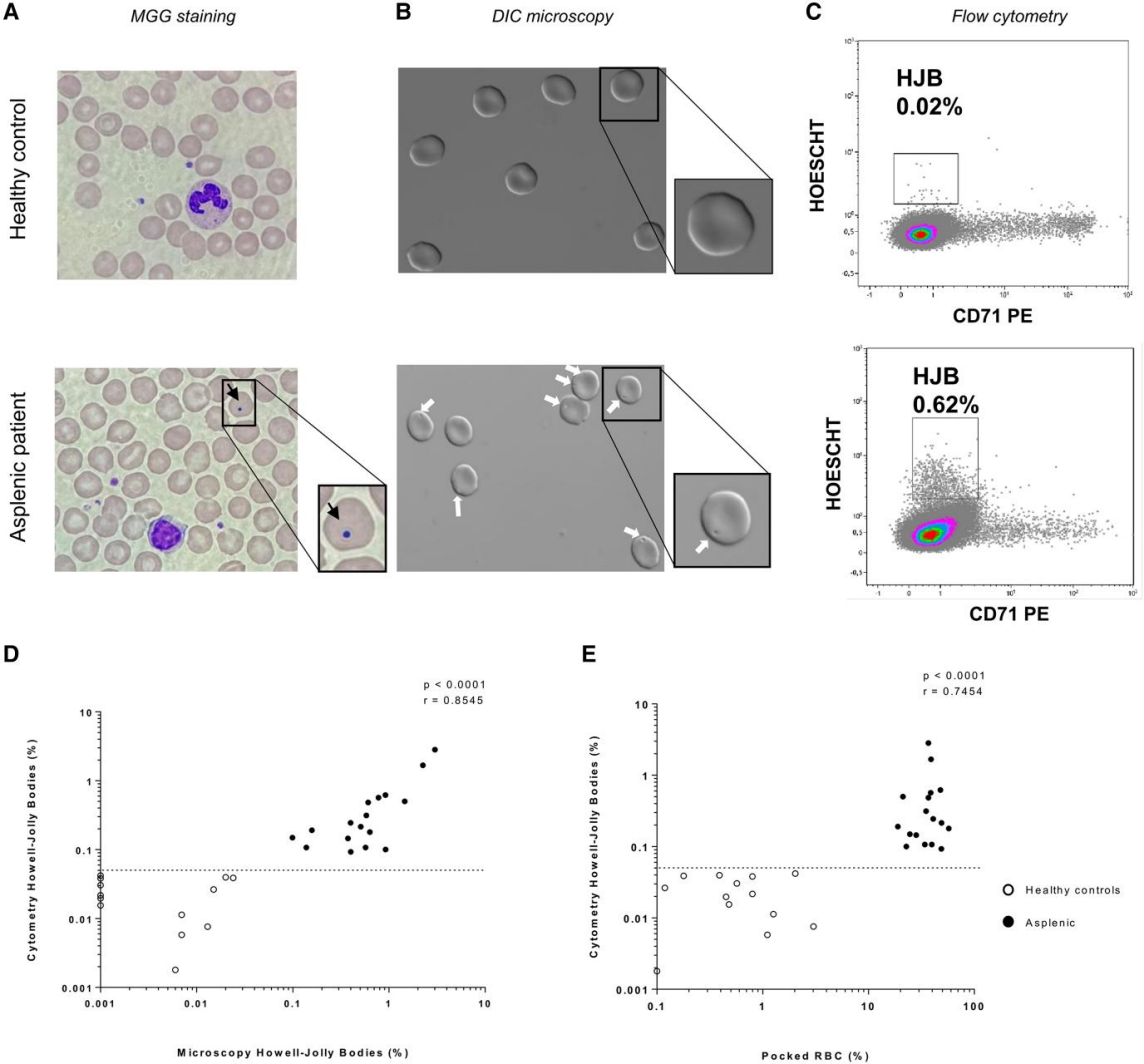
Validation of flow cytometry method to quantify Howell-Jolly bodies. *A*, May-Grunwald Giemsa staining of blood cells from healthy controls and asplenic patients. The black arrow indicates red blood cells containing HBJs. *B*, DIC microscopy imaging of red blood cells from healthy controls and asplenic patients. The white arrow indicates pocked cells. *C*, Flow cytometry plot representing red blood cells containing HJBs. *D*, Comparison between MGG microscopy and flow cytometry. n = 13 healthy controls (white circle); n = 17 asplenic patients (black circle). In healthy controls, values equal to 0 have been arbitrarily set to 0.001. *E*, Comparison between percentage of pocked RBCs and percentage of HJBs by flow cytometry. n = 13 healthy controls (white circle); n = 17 asplenic patients (black circle). In healthy controls, values equal to 0 have been arbitrarily set to 0.1. Correlations were calculated with Spearman’s correlation test. Abbreviations: DIC, Differential Interference Contrast; HJBs, Howell-Jolly bodies; MGG, May-Grunwald Giemsa; RBCs, red blood cells.

We established a cytometry protocol to study the filtration and immunological functions of the spleen, with the advantage of rapidly and reliably quantifying HJBs and MZBs in a large number of mature erythrocytes. HJB results obtained by cytometry and microscopy (Figure 1*A*–*C*) were compared and showed a strong correlation (r = 0.8545; *P* < .0001) (Figure 1*D*), and a moderate correlation was demonstrated between HJBs and pocked RBCs (r = 0.7454; *P* < .0001) (Figure 1*E*). Using Cohen's kappa coefficient, the agreement appeared perfect (κ = 1) between the HJBs using cytometry and microscopy, and it was perfect (κ = 1) between HJBs by cytometry and pocked RBCs. An HJB count >0.15% to 1% has been reported to be indicative of a loss of splenic filtration function [18] [22]. Pocked RBCs generally account for <4% of RBCs in healthy subjects, and asplenia is defined by the presence of >12% of pocked RBCs [18, 23]. ROC curves were established to evaluate the capacity of the test to distinguish splenectomized patients from controls (Supplementary Figure 1). Based on ROC curves in healthy donors and asplenic patients and literature data, we determined an optimal cutoff value of 0.05% for HJBs with 100% sensitivity and specificity >92% (AUC = 1). For pocked RBCs, a value of 11% offered the best discrimination between the asplenic patients (mean value, 36.5%) and healthy controls (mean value, 0.86%) with 100% sensitivity and specificity.

**Figure 2.**
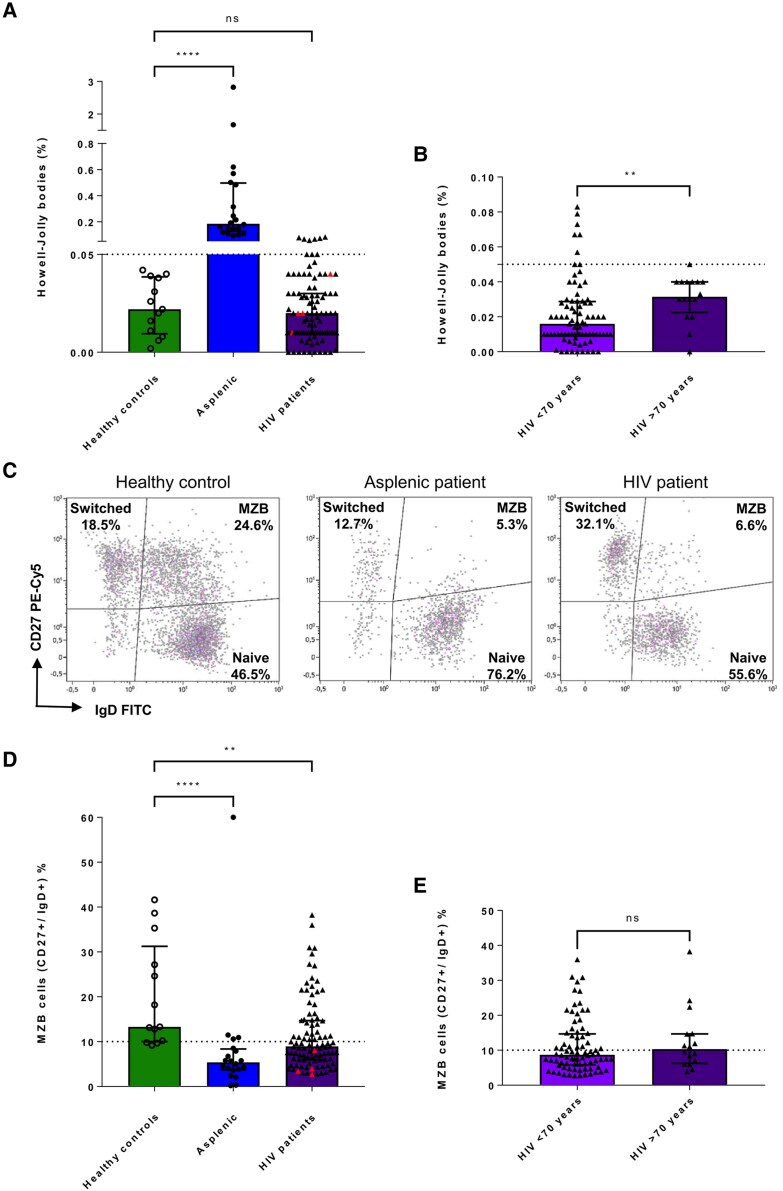
Evaluation of splenic filtration and immune functions. *A*, Quantification of Howell-Jolly bodies by flow cytometry. n = 13 healthy controls (left bar); n = 20 asplenic patients (middle bar); n = 96 HIV patients (right bar). Red triangle: *S. pneumoniae*+. The gray line represents the threshold. Significance was determined by Kruskal-Wallis test (*P* < .05). *B*, Quantification of Howell-Jolly bodies in HIV patients. n = 70<70 years (left bar); n = 16–>70 years (right bar). The gray line represents the threshold. Significance was determined by Mann-Whitney test (*P* < .05). *C*, Flow cytometry plots representing MZB cells in healthy controls, asplenic patients, and HIV patients. *D*, Quantification of marginal zone B cells CD27+ IgD+ by flow cytometry. n = 13 healthy controls (left bar); n = 20 asplenic patients (middle bar); n = 96 HIV patients (right bar). Red triangle: *S. pneumoniae*+. The gray line represents the threshold. Significance was determined by Kruskal-Wallis test (*P* < .05). *E*, Quantification of memory B cells CD27+ IgD+ in HIV patients. n = 70 <70 years (left bar); n = 16–>70 years (right bar). The gray line represents the threshold. Significance was determined by Mann-Whitney test (*P* < .05). Abbreviations: IG, immunoglobulin; MZB, marginal zone B cells.

An increase in HJBs was observed in asplenic participants (mean value, 0.433%) compared with healthy donors (mean value, 0.023%; *P* < .0001) (Figure 2*A*). Proportions of MZBs were decreased in asplenic participants (mean value, 7.96%) compared with healthy donors (mean value, 20.28%; *P* < .0001) (Figure 2*C*, *D*). Based on data from the literature [24] and ROC curves in healthy donors and asplenic patients, the optimal threshold for the best diagnostic performance was 10% of MZB cells with 80% sensitivity and 77% specificity (AUC = 1) (Supplementary Figure 1).

### Persons With HIV

A total of 96 participants on prolonged ART were included in the study. Among them, 16 were aged >70 years, and 4 had an history of invasive pneumococcal disease. Participants’ characteristics are summarized in Table 1. The median age of the participants was 55 years. The mean duration of HIV suppression was 11.5 years.

### Splenic Filtration Assessed by Erythrocytes Containing HJBs in PWH on Prolonged ART

We quantified HJBs (CD45–, CD235a+, CD71–, Hoescht+) using flow cytometry in 96 HIV patients compared with healthy controls and asplenic patients. We observed an impaired splenic filtration capacity defined by number of HJBs >0.05% of erythrocytes in 9 PWH (9.4%) (Figure 2*A*). Only 1 participant in the group of PWH over 70 had HJBs over the cutoff value. HJB frequency was higher in the group of PWH aged >70 compared with the younger group (mean value, 0.031% and 0.021%, respectively; *P* = .01) (Figure 2*B*). The mean HJB frequency was 0.023% in both PWH and healthy controls, indicating no significant difference in red pulp function between the groups (Figure 2*A*). None of the 4 participants with a history of invasive pneumococcal disease had an abnormal HJB count.

We assessed factors associated with the presence of HJBs. A history of AIDS (Centers for Disease Control and Prevention class C) was associated with the presence of HJBs above the threshold (OR, 6.6; 95% CI, 1.714–25.17). There was no difference in CD4 or CD8, lymphocytes, CD4/CD8 ratio, or CD4 NADIR in HIV patients associated with a value of HJBs >0.05% erythrocytes (Table 2). We did not observe any association between gender and HJB or MZB cells (data not shown). Linear regression analyses of continuous variables did not yield significant results (Supplementary Table 1).

**Table 2.** Determinants of Hyposplenism in HIV Patients

Variable	HBJ ≥0.05% (n = 9)	HBJ <0.05% (n = 87)			MZB <10% (n = 57)	MZB ≥10% (n = 39)		
Continuous Variables: Normally Distributed	Mean (95% CI)	Mean (95% CI)	*P*		Mean (95% CI)	Mean (95% CI)	*P*	
Age, y	55 (46–64)	56 (53–58)	.*930*		55 (52–59)	56 (51–60)	.*9401*	
Continuous Variables: Non–Normally Distributed	Median (IQR)	Median (IQR)	*P*		Median (IQR)	Median (IQR)	*P*	
Lymphocytes, number/mm^3^	2305 (1699–3187)	1834 (1332–2344)	*.0808*		1850 (1346–2403)	1883 (1395–2296)	*.9099*	
CD4, number/mm^3^	779 (608–941)	652 (436–829)	*.136*		647 (431–832)	700 (532–868)	*.423*	
CD4/CD8 ratio	1 (0.55–1.7)	1 (0.7–1.5)	*.750*		1 (0.7–1.5)	1 (0.7–1.5)	*.975*	
Binary Variables	n/N (%)	n/N (%)	OR (95% CI)	*P*	n/N (%)	n/N (%)	OR (95% CI)	*P*
AIDS-defining condition (CDC class C)	6/9 (66.7)	20/86 (23)	**6.6 (1.7–25.2)**	***.012***	17/56 (30.3)	9/39 (23.1)	0.94 (0.37–2.23)	…
Pneumoccocal disease	0/6 (0)	4/90 (4.6)	…	…	4/57 (7.1)	0/39 (0)	∞ (0.66 to ∞)	…
NADIR ≤200 CD4 cells/mm^3^	…	…	…	…	31/57 (54.4)	13/39 (33.3)	**2.38 (1.05–5.27)**	*.06*

Values in bold indicate OR that excludes 1 or *P* < .05. *P* values are indicated in italic. Mean ages were compared using a t-test; other continuous variables were compared using the Mann-Whitney *U* test.

Abbreviations: CDC, Centers for Disease Control and Prevention; HJB, Howell-Jolly body; IQR, interquartile range; MZB, marginal zone B; OR, odds ratio.

### Decrease of Marginal Zone B Lymphocytes in HIV Patients

To analyze immune splenic function, we quantified marginal zone B lymphocytes (CD19+, CD27+, IgD+). A decrease in CD27+/IgD+ B cells population was observed in PWH compared with healthy donors (*P* = .01), with 57 patients (59.4%) presenting a depletion of MZBs (<10% of LB), including the 4 participants with a history of invasive pneumococcal disease (Figure 2*C, D*).

In HIV patients aged >70 years, the frequency of splenic marginal LB was comparable to that of other participants (Figure 2*E*). We did not find any association between CD4 or CD8, lymphocytes, NADIR CD4, CD4/CD8 ratio, or AIDS and MZB lymphocyte depletion (Table 2). We did not observe any association between gender and HJB or MZB cells (data not shown). Linear regression analyses evaluating factors associated with MZB percentages did not yield significant results (Supplementary Table 1).

We did not find a correlation between MZB and HJB percentage in PWH. Among the 9 participants with HJBs >0.05%, 5 patients had also a depletion in MZBs.

## DISCUSSION

This study explores spleen function in PWH on prolonged ART. Our findings underscore the need for biological assessments of both the filtration and immunological function of the spleen in this population, as discrepancies may exist between these 2 splenic functions. Splenic filtration function was largely intact in the majority of PWH on prolonged ART, as <10% of the participants exhibited HJBs. While hyposplenism is present in a subset of PWH on prolonged ART, it is not as prevalent as before the advent of ART, when HJBs were observed in up to 36% of PWH [3]. The association between a history of AIDS and elevated HJB levels suggests that severe immunosuppression, particularly in the advanced stages of HIV, may have long-lasting effects on splenic function despite subsequent viral suppression with ART. Our results are consistent with the study by Bauer et al., which reports a prevalence of HJBs of 9.9% and suggests that HJBs are associated with the clinical stage of the disease and may be reversed by successful ART [4]. Interestingly, even among participants with a history of invasive pneumococcal disease, none exhibited abnormal HJB counts in our study. It is notable, however, that the frequency of HJBs was higher in older HIV patients (>70 years), although only 1 individual in this age group had HJB levels above the pathological threshold. Numerous studies have demonstrated that aging adversely affects the splenic function in mice [25]. In humans, age-related changes of the microarchitecture of the spleen have also been described [26].

In contrast to the relative preservation of filtration function, our study found a significant depletion of MZBs in PWH. Nearly 60% of PWH exhibited low levels of these cells (<10% of B lymphocytes), indicating compromised immune function. This depletion was observed in participants who had been on prolonged ART, suggesting that the immunological aspect of splenic function may be more susceptible to long-term damage from HIV infection than the filtration function. The causes of MZB depletion remain largely unknown, but gut injuries likely play a key role in this phenomenon.

The spleen is immunologically linked with the gut, and during hyposplenism associated with gastrointestinal disorders and HIV infection, spleen structural disorganizations that lead to the alteration of B-cell follicles are observed [19]. MZB cells occupy distinct spatial niches within both the spleen and gut-associated lymphoid tissues (GALTs), reflecting a functional compartmentalization of systemic and mucosal immune responses [27]. Early and persistent damage to GALTs in HIV infection may therefore impair splenic MZB-cell homeostasis through disrupted immune crosstalk between these compartments. Notably, a subpopulation of MZB cells expresses higher levels of the gut-homing marker β7 integrin, reinforcing the hypothesis that GALT dysfunction could contribute to MZB depletion [28]. HIV targets and depletes CD4+ T cells within these tissues [29]. The disruption of the cellular microenvironment in GALT contributes to the breakdown of the mucosal barrier, allowing microbial translocation that fuels chronic immune activation [30]. This early damage to GALT is a hallmark of HIV pathogenesis and may lead to splenic atrophy and architectural changes in the spleen, disrupting the microenvironment necessary for MZB-cell survival and function. In our study, MZB depletion was not different between participants aged over or under 70. In aged mice, the reduced number of MZBs is associated with impaired T-independent antibody responses and decline in the spleen's capacity to mount effective immune reactions against encapsulated bacteria [31]. In humans, a decline of MZBs with aging has been inconsistently observed, with 1 study reporting significant reductions and another showing minimal or no change [32, 33].

The lack of correlation between MZB depletion and HJB history suggests that different factors drive the impairment of red pulp and white pulp functions. The differential impact of age and HIV infection on splenic filtration and immune function has important clinical implications. While ART appears to mitigate some aspects of splenic dysfunction, particularly filtration, the persistent depletion of MZB suggests that PWH may remain at heightened risk for certain infections, despite achieving viral suppression.

This finding highlights the need for spleen function analysis in clinical settings, especially in older HIV patients and those with a history of severe immunosuppression. The results of our study support the use of flow cytometry as a practical and reliable method for this purpose. Our findings helped to establish a threshold for detecting MZB cells and HJBs, which are not yet well defined in the literature. The strong correlation between HJB enumeration by cytometry and traditional microscopy, along with better analytical sensitivity and the ability to simultaneously assess MZB levels, makes this approach well suited for routine diagnostics and monitoring in PWH.

This study has several limitations. The relatively small sample size, particularly in the group of PWH aged >70, may limit the generalizability of our findings, and as such, the lack of association between age and MZB depletion or HJBs needs to be confirmed. The absence of immunological correlates in PWH with elevated HJBs may reflect persistent monocyte/macrophage dysfunction, which was not assessed in this study and should be explored in future investigations.

Additionally, the cross-sectional design precludes conclusions about the causality of the observed associations. Longitudinal studies with larger cohorts are needed to confirm these results and explore the long-term trajectory of splenic function in PWH on prolonged ART.

In conclusion, our study demonstrates that while splenic filtration function is relatively preserved in PWH on prolonged ART, significant impairments in immune function persist, as evidenced by the depletion of MZBs. These findings underscore the need for assessments of splenic function in PWH. Understanding splenic impairments in PWH is crucial for developing interventions aimed at preserving or restoring immunological and filtration function of the spleen.

## Supplementary Material

ofaf644_Supplementary_Data

## Supplementary Data

Supplementary materials are available at *Open Forum Infectious Diseases* online. Consisting of data provided by the authors to benefit the reader, the posted materials are not copyedited and are the sole responsibility of the authors, so questions or comments should be addressed to the corresponding author.
