# Supplementary material for: Distinct Defects in Marginal Zone B Cells and Filtration Function Characterize Hyposplenism in Persons With HIV-1 on Prolonged ART
Source: Open Forum Infect Dis. 2025 Oct 14;12(11):ofaf644. doi: 10.1093/ofid/ofaf644 (PMC12616002; doi:10.1093/ofid/ofaf644)
Supplement: ofaf644_Supplementary_Data [file ofaf644_supplementary_data.zip › DEBIESSE_Hyposplenism_in_PWH_SUPP_DATA.docx]

**SUPPLEMENTAL DATA**

**Supp. Table S1: Linear regression analysis for determinants of Hyposplenism in HIV patients (n=96)**

| **Variable** |  | **HBJ** | |  | **MZB** | |
| --- | --- | --- | --- | --- | --- | --- |
| **Continuous variables: Normally distributed** | **Median (IQR)** | **R square** | **P** |  | **R square** | **P** |
| Age (years) | 55 (22-64) | 0.02243 | 0.1453 |  | 0.0033 | 0.577 |
| **Continuous variables: Non-Normally distributed** | **Median (IQR)** | **R square** | **P** |  | **R square** | **P** |
| CD4 number / mm3 | 674 (454.5-844.8) | 0.06415 | **0.0128** |  | 0.0001 | 0.940 |
| CD4/CD8 ratio | 1 (0.7-1.5) | 0.00185 | 0.6774 |  | 0.0075 | 0.401 |
| CD4 cell count NADIR | 215.3 (83-361.9) | 0.01214 | 0.2877 |  | 0.0175 | 0.201 |
| Values in bold indicate P < 0.05; IQR: interquartile Range. | | | | | | |

**Supp. Figure S1: Receiver operating characteristics (ROCs) curve analysis for determining optimal cut-off for Howell-Jolly Bodies, RBC-pocked and Marginal Zone B cells.**

**A:** ROC curve analysis of the quantification of HJB by microscopy (light blue line). **B:** ROC curve analysis of HJB quantification by flow cytometry (dark blue line). **C:** ROC curve analysis of quantification of pocked cells by microscopy (green line). **D:** ROC curve analysis of quantification of MZB cells by flow cytometry (black line). AUC are indicated in the graph; Red point represented the optimal cut-off and value in red indicated the best discriminant threshold define use a contingency table of sensitivity/specificity at different threshold (13 healthy controls and 17 asplenic patients).
