# Supplementary figures and images for: Distinct Defects in Marginal Zone B Cells and Filtration Function Characterize Hyposplenism in Persons With HIV-1 on Prolonged ART
Source: Open Forum Infect Dis. 2025 Oct 14;12(11):ofaf644. doi: 10.1093/ofid/ofaf644 (PMC12616002; doi:10.1093/ofid/ofaf644)

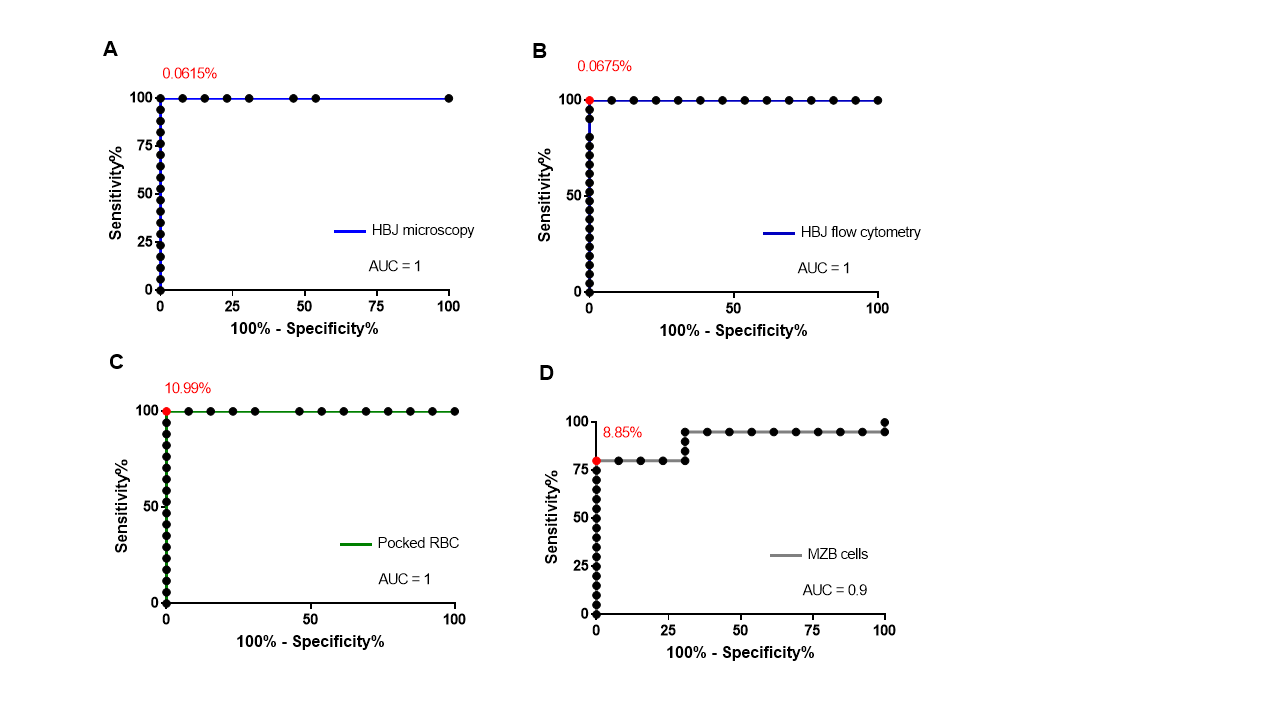

Supplement: ofaf644_Supplementary_Data [file ofaf644_supplementary_data.zip › supp.tif]
